# An Immortalized Genetic Mapping Population for Perennial Ryegrass: A Resource for Phenotyping and Complex Trait Mapping

**DOI:** 10.3389/fpls.2018.00717

**Published:** 2018-05-31

**Authors:** Janaki Velmurugan, Dan Milbourne, Vincent Connolly, J. S. Heslop-Harrison, Ulrike C. M. Anhalt, M. B. Lynch, Susanne Barth

**Affiliations:** ^1^Crops Environment and Land Use Programme, Oak Park Research Centre, Teagasc – The Irish Agriculture and Food Development Authority, Carlow, Ireland; ^2^School of Agriculture and Food Science, University College Dublin, Dublin, Ireland; ^3^Department of Genetics and Genome Biology, University of Leicester, Leicester, United Kingdom

**Keywords:** perennial ryegrass, *Lolium perenne*, recombinant inbred lines (RIL), genotyping by sequencing, mapping population, phenotyping

## Abstract

To address the lack of a truly portable, universal reference mapping population for perennial ryegrass, we have been developing a recombinant inbred line (RIL) mapping population of perennial ryegrass derived via single seed descent from a well-characterized F2 mapping population based on genetically distinct inbred parents in which the natural self-incompatibility (SI) system of perennial ryegrass has been overcome. We examined whether it is possible to create a genotyping by sequencing (GBS) based genetic linkage map in a small population of the F6 generation of this population. We used 41 F6 genotypes for GBS with *PstI/MspI*-based libraries. We successfully developed a genetic linkage map comprising 6074 SNP markers, placing a further 22080 presence and absence variation (PAV) markers on the map. We examined the resulting genetic map for general and RIL specific features. Overall segregation distortion levels were similar to those experienced in the F2 generation, but segregation distortion was reduced on linkage group 6 and increased on linkage group 7. Residual heterozygosity in the F6 generation was observed at a level of 5.4%. There was a high proportion of chromosomes (30%) exhibiting the intact haplotype of the original inbred parents of the F1 genotype from which the population is derived, pointing to a tendency for chromosomes to assort without recombining. This could affect the applicability of these lines and might make them more suitable for situations where repressed recombination is an advantage. Inter- and intra-chromosomal linkage disequilibrium (LD) analysis suggested that the map order was robust. We conclude that this RIL population, and subsequent F7 and F8 generations will be useful for genetic analysis and phenotyping of agronomic and biological important traits in perennial ryegrass.

## Introduction

Genetic linkage mapping in segregating populations is one of the classic approaches to gain insights into the genetic control of key characteristics in a species, and can also form the basis of molecular marker-assisted selection in plant breeding. In perennial ryegrass, over the previous two decades, numerous genetic linkage mapping studies have been carried out, with a host of different mortal populations used to map a range of quantitative traits (summarized in a QTL meta-analysis by Shinozuka et al., 2012).

To date mapping populations in perennial ryegrass have all been temporary population types, such as F1 populations – generated from a cross between two parental genotypes of contrasting characters, F2 populations -obtained by self-pollination of the F1, and BC populations -where the F1 individual is crossed with one of the parents of the F1 (Bert et al., 1999; Armstead et al., 2002; Jensen et al., 2005; Muylle et al., 2005; Anhalt et al., 2008) These populations due to the allogamous species character and by being highly heterozygous cannot be propagated indefinitely and easily distributed through seed. DNA samples from the populations are rarely available, and many characters of interest have remained unscored.

Seed propagated, immortalized populations for perennial ryegrass would be very useful for trait dissection. The commonly used immortalized mapping population types include RILs (produced by self-pollinating the F2 individuals to a certain number of generations), and doubled haploid (DH) populations (produced by artificially doubling the chromosomes of the haploid material). DH populations are less time consuming to generate but require well established protocols to work for particular species. Generation of RILs is time, labor and cost intensive. However, in addition to immortalization, RILs offer an advantage in that the additional number of recombination events experienced per genotype during recurrent rounds of self-pollination/inbreeding required to produce them increases the resolving power of the resulting mapping populations and allows fine mapping of QTL with fewer individuals (Takuno et al., 2012). Genetic maps using RIL populations have been generated for numerous different species including minor cereals like pearl millet (Rajaram et al., 2013) and oat (Huang et al., 2014). In addition to increasing the genetic map resolution for detecting QTLs, RILs are fixed and thereby provide an immortalized reference mapping population which can be genotyped once and can be phenotyped multiple times for different traits under different environments.

Perennial ryegrass is an obligate outbreeder and has a genetically determined self-incompatibility system (SI) (Thorogood et al., 2002). The Irish perennial ryegrass breeding program of Teagasc has developed germplasm in which the self-incompatibility system has been broken down and two inbred lines from this material were used to generate the ‘F2 Biomass mapping population’ that has been the basis of several genetic mapping studies phenotyping traits like SI, non-polar metabolites, crown rust resistance and biomass yield (Anhalt et al., 2008, 2009; Tomaszewski et al., 2010, 2012; Foito et al., 2015). Most recently, the population was used to develop a high density, single nucleotide polymorphism (SNP)-based map based on genotyping by sequencing (GBS) in which reads were aligned to a reference assembly generated by Illumina shotgun sequencing of the paternal grandparent (Velmurugan et al., 2016).

This ‘F2 Biomass mapping population’ based on ‘cv S24’ by ‘cv Premo’ parental origins has also been used as the starting point for the production of a RIL population via single seed descent, with the ultimate goal of producing a immortalized reference mapping population which could be shared across different research groups. A small collection from the F6 generation of RILs was used to construct a genetic linkage map using a similar GBS-based approach as adopted in Velmurugan et al. (2016) to (1) investigate the genomic and genetic constitution of the RILs in terms of features such as recombination rate, (2) the level of heterozygosity, (3) chromosomal blocks inherited by identity by descent, (4) genome wide linkage disequilibrium (LD) patterns, and (5) segregation distortion.

## Materials and Methods

### Plant Material

A set of 41 individuals from the F6 generation of an ongoing initiative at Teagasc to construct a RIL population for perennial ryegrass was used in this study. The lines were produced by single seed descent from individuals of the F2 Biomass population (Anhalt et al., 2008, 2009; Tomaszewski et al., 2012).

Both parents of the F1 generation were inbred lines that were a by-product from the development of cytoplasmic male sterile lines (CMS) and were the maintainer lines of this CMS program (Connolly and Wright-Turner, 1984). These maintainer lines were self-pollinated (S) for up to seven generations. The distinct germplasm pools from which these maintainer lines were developed were the cultivars ‘S24’ (IGER, Wales/United Kingdom) in the case of the maternal inbred parent, and ‘Premo’ (Mommersteeg International BV, Netherlands) in the case of the paternal inbred parent.

**FIGURE 1:**
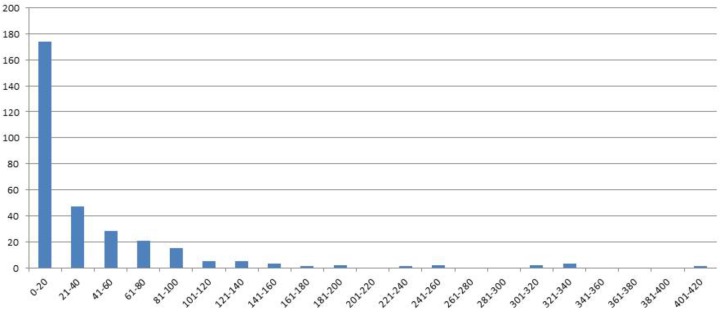
Distribution of genotype classes (*x*-axis) according the number of seeds (*y*-axis) for F4 generation seed data, harvest year 2007.

The maternal parent was emasculated under a binocular microscope and stigmas were pollinated with pollen from the paternal plant. Pollinated florets were bagged in cellophane bags and individual F1 seed was harvested. F1 seed was planted and a single F1 plant selected for self-pollination. This F1 plant was vegetatively multiplied to produce multiple clones, and the clones of this F1 plant were self-pollinated by applying cellophane pollination bags prior to ear emergence. Bags were shaken twice daily to ensure a good propagation of the pollen cloud for the self-pollination. The resulting F2 seed was harvested, planted and over 300 independent lines were grown up. Each individual of this F2 population was self-pollinated as previously described. Seeds were harvested from each independent F2 plant and the F3 seed was cleaned for each of the individual F3 bulks. F3 seed were then sown and one single plant of each family was selected for self-pollination in the following cycle of single seed descent multiplication (**Figure 1**). All other independent seed lines from the same family were discarded. Chosen individuals from each family for each generation were selected to be non-albino or non-variegated and not too dwarf to ensure that it was still possible to obtain seed yield from these plants. According to this procedure, independent recombinant inbred lines (RILs) were generated to generation 6. Seed yield was recorded from the F3 to the F6 lines.

### GBS Libraries Construction

The GBS libraries were constructed with the two enzyme approach (Poland et al., 2012). The genomic DNA was co-digested with restriction enzymes *Pst*I (CTGCAG) and *Msp*I (CCGG) and the barcode adaptors (Supplementary Table 1) varying in length from 4 to 8 base pairs (bp) were then ligated to the individual samples and polymerase chain reaction-amplified. Several genotypes had more than one GBS library produced (Supplementary Table 1). GBS libraries with insert sizes of 100–300 bp were sequenced on a single sequencing lane of Illumina HiSeq 2000 for 100 bp paired end reads.

### Variant Calling Pipeline

Illumina’s CASAVA software (Hosseini et al., 2010) was used for de-multiplexing the samples. To remove the low quality bases at the end, the de-multiplexed reads were trimmed to 66 bp (for phred score value of greater than 20) using fastx_trimmer (Catchen et al., 2013). The trimmed reads were then aligned to the *Lolium* gene space reference assembly consisting of 424,750 scaffolds (Velmurugan et al., 2016) using the Bowtie alignment program (Langmead et al., 2009). The accepted alignment parameters include (i) allowing two mismatches (-v 3) between the reference and the individual sample (ii) suppressing alignments for reads mapping to more than one position in the genome (-m 1). The alignment results were produced in SAM format and SAMtools-0.1.18 (Li et al., 2009) was used to post process the alignment files (converting SAM to BAM, sorting, indexing) and SAMtools mpileup command was used to create a consensus mpileup file. From the consensus mpileup file, the SNP variants were called using VarScan.v2.2.11 (Koboldt et al., 2009) with the default settings (minimum read depth of 8; minimum average phred quality of 20, variant allele frequency of 0.2 and minimum average quality of 20).

From the variant calling results obtained using VarScan.v2.2.11, SNP filtering was performed using a custom awk script to extract markers that could be used for genetic mapping purposes. This was done by applying two filters (i) excluding obvious monomorphic markers (by filtering the markers for presence of either parental allele or for both alleles) and (ii) excluding markers with missing data (>35%).

A single merged bam file generated by using the SAMtools merge command from all the individual alignment files was used to identify presence/absence variants (PAVs). First, the merged bam file was converted to SAM format and then a simple two-step filtering approach as described in Velmurugan et al. (2016) was used to identify potential PAVs. The paired end sequencing provided additional read depth coverage at a position to call PAVs. Reads that were mapped on both the strands (SAMtools flag 99 pairing with 147; 83 pairing with 163) were used for the read depth count. The third (RNAME: reference sequence name) and fourth (POS: 1-based leftmost mapping position) field in the alignment file was used in conjunction to estimate the unique positions in the genome that has alignments. Data were then filtered for a 1:1 ratio and to allow segregation distortion, ±20% was included on either side of the expected segregation ratio, and possession of an average read depth of 8 at each position in the genome for individuals exhibiting alignments.

### Genetic Linkage Map Construction

After filtering the SNP and PA markers using the above mentioned filters, the SNP and PA markers were carried forward for constructing a genetic linkage map. The map construction strategy described in Velmurugan et al. (2016) was applied for this dataset.

The markers were first separated into linkage groups using R/qtl (Broman et al., 2003). Then JoinMap4.1 (Van Ooijen, 2011) was used for further map calculations. R/qtl was used instead of Joinmap 4.1 initially because of the large number of markers involved. The computational time of grouping the markers into linkage groups in R/qtl is quite short compared to using JoinMap4.1 for this step of the analysis.

The SNP markers were genotype coded as F2 population type and imported into R/qtl using read.cross() function. The est.rf() function was used to estimate the pairwise recombinant fractions between the markers and formLinkageGroups() function was used for grouping the markers into linkage groups. The scaffold names anchored with the genetic markers in the F2 population (Velmurugan et al., 2016) was used for assigning the linkage groups to chromosomes in the F6 RIL population since the same reference genome was used in both studies.

After the markers were assigned to the respective chromosome, the marker genotype data from each linkage group was exported from R/qtl using write.cross() function. Then for each chromosome, a JoinMap 4.1 locus genotype file coded as RIL population type was created. The locus genotype files created for each chromosome were loaded into JoinMap 4.1 and analyzed further for map calculations.

The default Joinmap 4.1 calculation options for the pairwise distance measures were adopted and markers were grouped using independence logarithm of odds (LOD) function. The markers that grouped at a LOD range of [6–9] were carried forward for further map calculations. The grouping function in JoinMap 4.1 places the redundant loci in bins and only the non-identical loci are carried forward. The initial map order was calculated for this non-redundant dataset using the maximum likelihood map algorithm implemented in JoinMap 4.1.

To deal with map inflation caused by the effect of low levels of genotyping error, we used the GBS error correction tool ‘PLUMAGE v. 2.0’ (Spindel et al., 2013) to correct any singletons. Using this python script, any singletons observed were replaced with missing values and a new JoinMap 4.1 locus genotype file was created from this error corrected dataset for each linkage group. The map order calculated based on this error corrected dataset using the maximum likelihood algorithm of JoinMap 4.1 to create a framework linkage map showed that in individuals retaining large heterozygous blocks, these blocks were interrupted by numerous apparent recombination events at a frequency far higher than would be theoretically possible given the number of meiosis events separating the homozygous grandparental donors and the F6 individuals. To attempt to ameliorate this, the map was recalculated by filtering the data (organized by linkage group) to retain only high stringent genotype calls (where a read depth of less than 20 were scored as missing) for the SNP markers.

The filtered high stringent SNP markers identified from the approach described above were used for constructing the framework genetic map. The same mapping strategy as adopted previously was applied. To place the PAVs on the framework linkage map, a series of shell scripting was performed on the pairwise data file exported from the JoinMap 4.1 analysis based on the recombinant fraction and LOD score values obtained using the framework SNP markers and the PAVs. The PAVs were placed on the framework map by assigning them to the map position of the framework SNP marker to which they exhibit the lowest recombinant fraction (RF) value and the highest LOD score.

### Segregation Distortion

To examine segregation distortion, we used the *p*-values (threshold of <0.05) associated with chi-squares (reported from the locus genotype frequencies results of the maximum likelihood map order of the final framework SNP markers) as an estimate for distortion exported from JoinMap 4.1. To examine the pattern of deviation toward the source alleles (grandpaternal/grandmaternal/heterozygous) along the chromosome, we used the number of genotypes scored for different alleles at each locus column values from the locus genotype frequencies table. The number of distorted framework SNP loci versus non-distorted per chromosome was used as a measure to quantify the overall level of distortion.

### RIL Specific Features: Heterozygosity Retention

To examine the level of heterozygosity retention in the F6 generation of RILs, we used the results from the ‘Locus genotype frequencies’ table exported from JoinMap 4.1 analysis. The percentage of heterozygosity per locus was obtained by dividing the number of genotypes that exhibit heterozygous alleles at each locus by the total number of individuals used in the study. This number was averaged across all the chromosomes to obtain the average heterozygosity per locus value for the F6 generation of RILs.

To examine the chromosomal segments derived by lineal descent, we used the genotype data of the common SNP markers between both the F2 and F6 maps (before error correction). The graphical genotypes of the loci were then ordered by the F2 map position to identify the segments derived by lineal descent from the F2 to the F6.

### RIL Specific Features: Crossover Frequency

The graphical genotypes of the final maximum likelihood map were used to count the recombination blocks observed per chromosome for the individuals present in the map: We used the map order information obtained from JoinMap, used conditional formatting in excel to color code the different alleles (a, h, b). The sum of these blocks across all the chromosomes was manually counted and were used as an estimate for total number of recombination events observed per individual. To avoid counting possible genotype errors as recombination events, the following rule was applied: a block of genotypes was considered a recombination event only when it was followed by at least three genotype calls.

The calculation used for estimating the expected total number of recombination counts per individual is as follows. In RILs, recombination occurs at every generation, but the ability to detect the number of crossovers reduces by half every generation (as it is masked by the increased accumulation of parental segments in each generation).

We used two calculations to get a theoretical expectation of the observable number of recombination events per individual for the F2 and the F6 generation. In the first instance we assumed one crossover per chromosome arm per meiosis. For perennial ryegrass at F1 generation there would be 14 crossovers observed per individual. As the observable number of crossovers reduces by half at each generation, there would be seven observable crossovers at F2, 3.50 at F3, 1.75 at F4, 0.87 at F5, and 0.43 at F6. By summing up the number of observable number of crossovers to the generation under interest gives the expected observable number of recombination events per individual for that generation, which is 21.0 and 27.5, respectively, for the F2 and the F6 generation. In an alternative method, based on that described in Dell’Acqua et al. (2015), we assumed a base genetic map length of 10 Morgan (1000 cM) for perennial ryegrass, equating to 10 recombination events per generation. Using this approach the expected number of recombination events was lower: 15.75 in the F2 generation and 21 in F6. We compared these values with the observed number of recombination events from the graphical genotypes results.

### Assessment of RILs Measured in Terms of Linkage Disequilibrium (LD)

To estimate the degree of LD across all the framework SNP markers on the map, we used the LD measure *r*^2^ calculated as the squared allele-frequency correlations using the TASSEL 5.0 bioinformatics analysis package (Bradbury et al., 2007). The LD plot function within TASSEL 5.0 was used to generate the heat map for LD. The pairwise recombinant fractions measured using *est.rf* function from R/qtl estimated for all pairs of framework SNP markers was used as a measure for recombinant fractions. *PlotRF* function was used to create a heat map for the estimated recombinant fractions and the LOD scores. These heat maps were used to visualize inter and intra chromosomal marker association.

## Results

### RILs Developed Across Generations and Years

Between 2005 and 2011 hundreds of RILs in varying numbers across the F3 to the F7 generation have been developed (**Table 1**).

**Table 1:** Number of RIL genotypes developed 2006–2011 (For the same generation the number of RILs was increased by repeating the selfings in consecutive years 2007–2011).

	2006	2007	2008	2010	2011
F3	311	71	53	94	67
F4		310	118	99	60
F5			222	99	134
F6				2	115
F7					80

In 2006 for the F3 generation 311 plants grew and the average of the number of seeds was 154. For the same generation the number of RILs was increased by repeating the selfings in 2007–2011. Genotypes were broken down in seed yielding classes of blocks of 20 genotypes to visualize seed yield. In the F4 generation in the 2007 harvest an average of 37 seeds from 310 individual genotypes was obtained. A minority of genotypes had greater than 160 seed (**Figure 1**).

**FIGURE 2:**
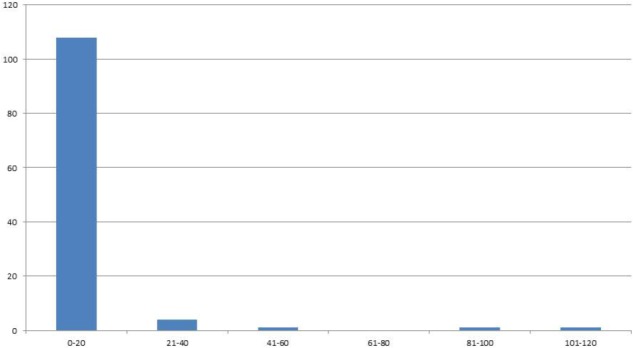
Distribution of genotypes into classes of 20 (*x*-axis) according the number of seeds (*y*-axis) for F6 generation seed harvested in 2011.

In the F4 generation, as in the F3, very few RIL families had higher seed yield compared to other families, and this trend was also seen in the F5 and F6 generations. In the F5 generation with 2008 seed we had a total of 222 individuals with an average of 24 seed (**Figure 2**). For the F6 generation in 2011 we had 115 individual genotypes and the average number of seed was six.

### A SNP-Based Map of the F6 Perennial Ryegrass RIL Population

**FIGURE 3:**
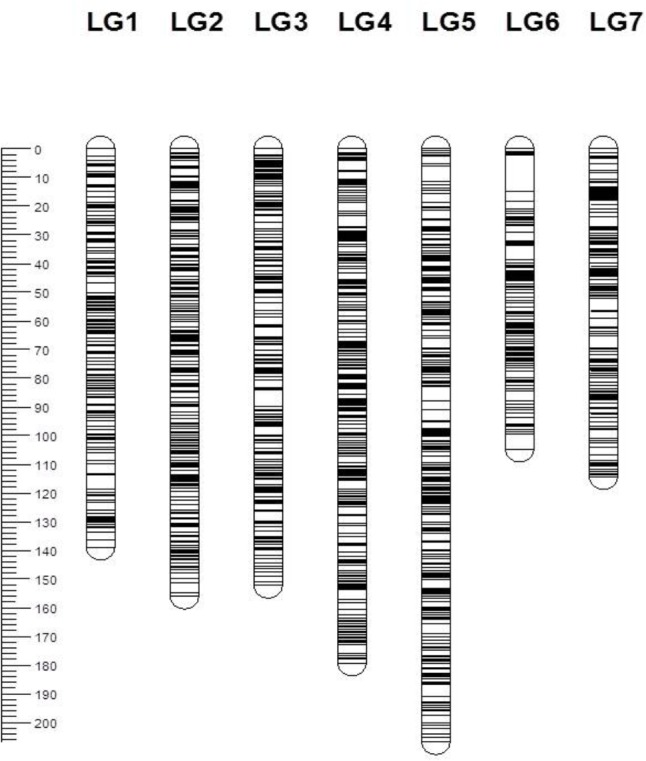
Genetic map of the 3,206 framework SNP markers ordered by map position for seven linkage groups. Each bar represents a linkage group and the line across the bars represents the map position of the SNP marker. The scale on the left represents the map position in centimorgans (cM).

**FIGURE 4:**
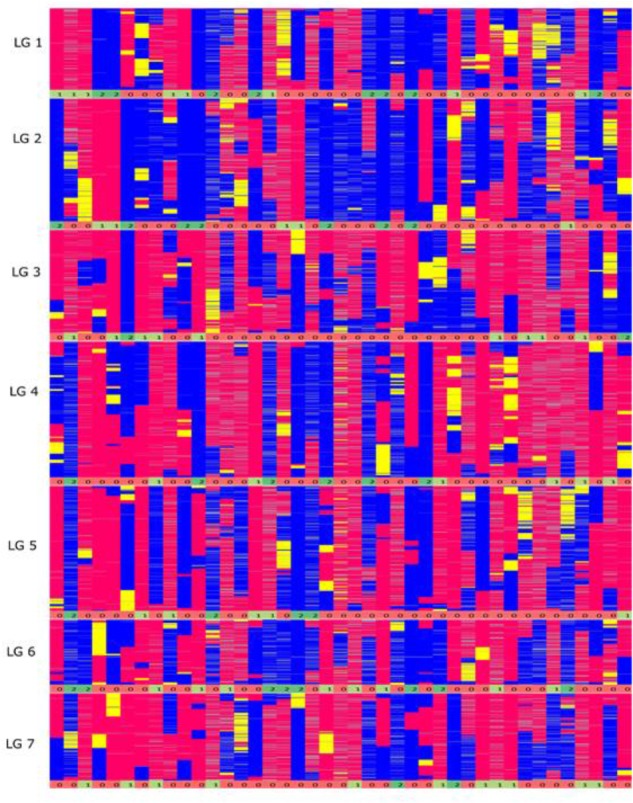
Graphical genotypes of the F6 framework SNP map. The *x*-axis represents the error corrected genotype calls of the 41 RIL individuals and the *y*-axis consist of markers ordered by chromosomal map position based on the final maximum likelihood map order of the framework SNP markers. The colors ‘pink’ represent homozygous alleles from the maternal parent, ‘blue’ represent homozygous alleles from paternal parent, ‘yellow’ as heterozygous alleles and gray as missing data. The numbers below each linkage group represents if the individual chromosome is non-recombinant/recombinant. Number 1 (color coded as yellow green) – indicates non-recombinant for maternal allele, 2 (color coded as green) – indicates non-recombinant for paternal allele and 0 (color coded as red) – indicates recombinant.

From a total of over 225 million reads, after alignment and filtering 6,072 high confidence SNPs were used to create the framework genetic linkage map. This represented 3,206 non-redundant loci on a total map length of 1,051.8cM (**Figure 3**). The filtering criteria utilized to identify the final set of high confidence SNPs is outlined in Supplementary Data File 2. A key step in the filtering process was to rely only on SNPs supported by a read depth of greater >20 in order reliably identify heterozygous genotypes. At lower read depths the map comprised large homozygous blocks interrupted by small heterozygous blocks at a higher than expected frequency. Utilizing the stringent read depth filtering criterion, we observed that heterozygous blocks became more coherent (**Figure 4**) suggesting lower confidence genotype calls due to lower read depth in previous versions of the map lacking this filtering step.

Presence–absence variants (PAVs) were placed on this framework map according to the method of Velmurugan et al. (2016). From the merged alignment SAM file, there were 479,748 independent alignment start positions. 39,244 segregating PAVs were identified and placed on the framework map. At a recombination fraction (RF) vs. LOD score threshold of 0.2/11-12, out of the total 39,244 PAVs identified, 22,080 resolved into independent seven linkage groups and were placed in bins defined by the 3206 framework SNP markers (**Table 2**). In total, 28,152 markers (22,080 PAVs and 6,072 SNPs) were placed on the final map representing 8,098 scaffolds in the assembly anchoring 294,918,024 bases (27%) of the total assembly (Supplementary Data File 2). Of the 8,098 scaffolds, 1,389 scaffolds (47,803,873 bp) had only SNP markers on them, 5,432 scaffolds (176,508,169 bp) had only PAV markers on them and 1,277 scaffolds (70,605,982) had both the SNP and PAV marker on them. Sequencing raw data have been submitted to NCBI Short Read Archive (SRA^1^) under project number SRP139320.

**Table 2:** Summary of the genetic map in terms of number of markers, marker type and genetic distance.

Linkage group	Total no. markers	No. of SNP bins (framework markers)	No. of SNP markers	No. of PAV markers	Map length (cM)	Average inter-marker distance (cM)	Maximum inter-marker distance (cM)
1	3,376	363	679	2,697	138.8	0.4	4.8
2	2,025	547	1,008	1,017	156	0.3	3.3
3	5,095	458	871	4,224	152.1	0.3	5.6
4	5,730	605	1,155	4,575	179.3	0.3	4.1
5	4,830	557	1,031	3,799	206.6	0.4	5.6
6	2,468	289	576	1,892	104.7	0.4	12.8
7	4,628	387	752	3,876	114.3	0.3	4.5
**Total**	28,152	3,206	6,072	22,080	1,051.8	0.3	NA

### Investigating the Robustness of the F6 Map

In order to investigate the robustness of the F6 map, we compared it to the SNP-based map of the F2 Biomass population (Velmurugan et al., 2016) from which the F6 population is derived, and also examined the presence of LD between and within linkage groups.

**FIGURE 5:**
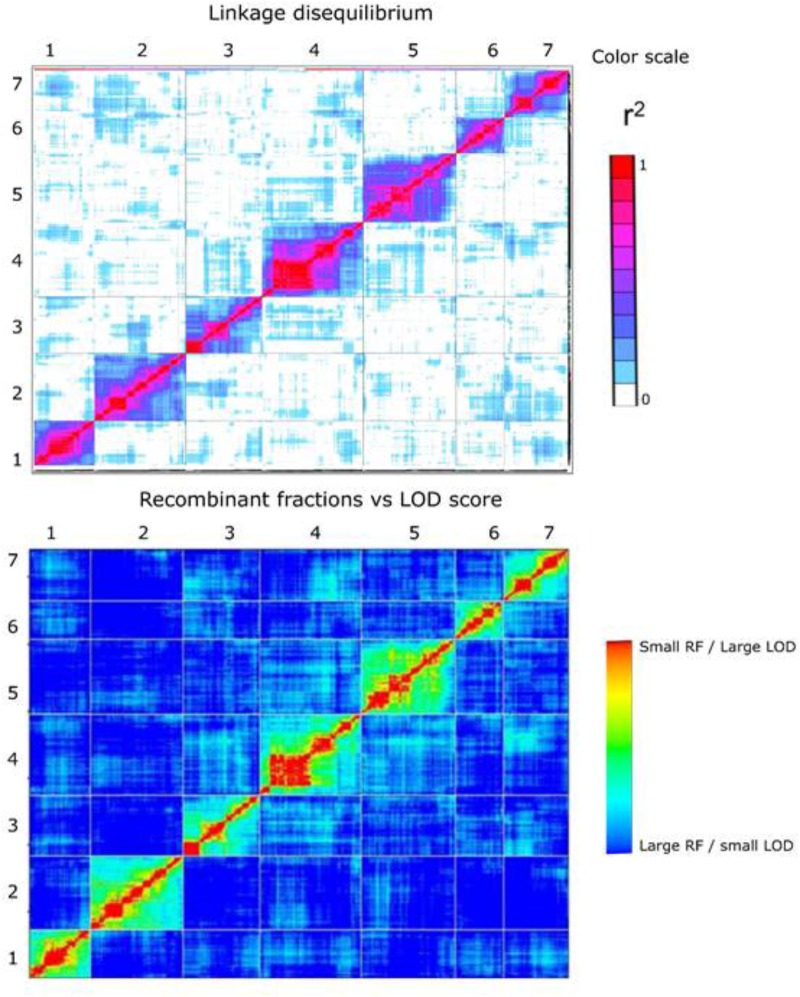
Heat map at the top represents linkage disequilibrium plot – pairwise *r*^2^ values (both upper-left and lower right triangle) measured using TASSEL 5.0 for all pairs of 3,206 framework SNP markers. The color scale ranges from white (no LD) to red (high LD). The heat map at the bottom represents plot of estimated recombination fractions (upper-left triangle) and LOD scores (lower-right triangle) for all pairs of markers. Red indicates linked (large LOD score or small recombination fraction) and blue indicates not linked (small LOD score or large recombination fraction). The number on the top and to the left of the heat map represents the linkage group.

In general, higher quality maps should exhibit relatively little intra- and inter-chromosomal LD unless there are specific selective forces causing this to be the case. We used the approach adopted by Rabbi et al. (2014) in the construction of ultra-high density map in cassava using the LD statistic *r*^2^ between all pairs of markers on the map as a measure to examine the extent of potentially spurious linkage between markers both between and within chromosomes. In plotted heat maps of all seven chromosomes comparing both pairwise *r*^2^ values from TASSEL, and also pairwise recombination fraction and LOD values from the mapping analysis in R/qtl a low level of off-diagonal association between marker pairs was found, indicating a generally robust marker order on the linkage map (**Figure 5**).

### Intergenerational Changes in the Distribution of Segregation Distortion During RIL Development

Out of the 3,206 non-redundant framework SNP markers 955 (30%) departed from the Mendelian segregation ratio of 1:1 (*p*-value < 0.05). This overall figure for the level of SD in terms of non-redundant framework SNP markers was effectively the same, dropping very slightly from 33% in the F2 Biomass population (Velmurugan et al., 2016) to 30% in the F6.

**FIGURE 6:**
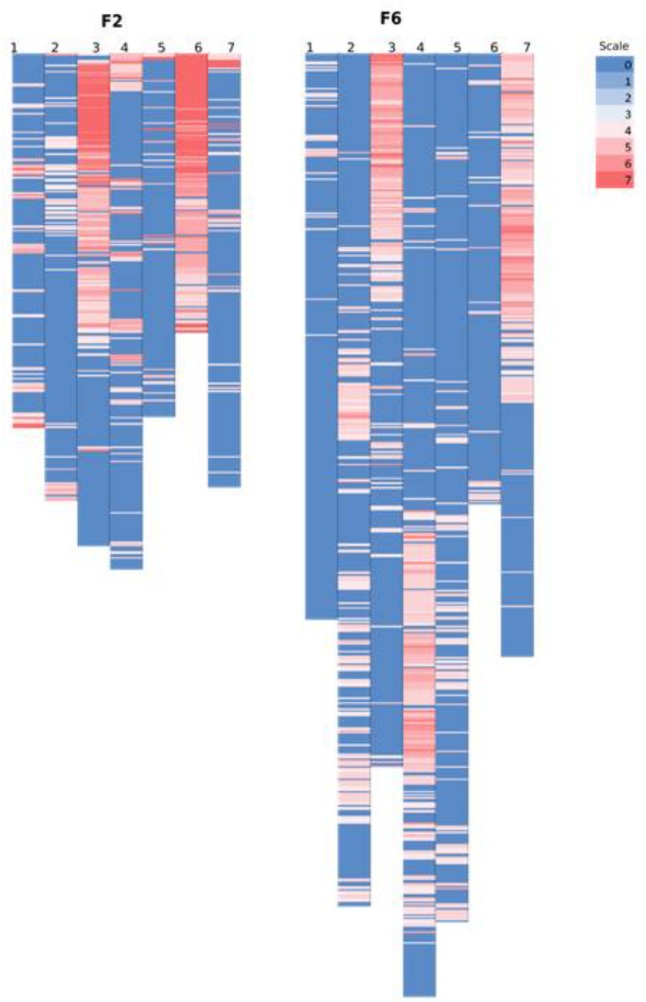
Comparing the location of segregation distortion regions along the chromosome in both the F2 and the F6 map. The level of distortion at each loci (*p*-values associated with the chi-square results from JoinMap 4.1 maximum likelihood final map calculation based on the framework SNP markers) are color scaled from blue (no distortion) to red (significant distortion – *p*-value ≤ 0.0000001). In the F2 map, the black text on the left bar along the chromosome denotes the position of the markers from the map of Anhalt et al. (2008).

However, on a per chromosome basis, a striking difference in terms of total percentage of framework distorted loci was observed for linkage group 6 (dropped from 96% in the F2 to 8% in the F6) and for linkage group 7 (increased from 15% in the F2 to 54% in the F6). Thus, linkage group 6 went from being the most distorted in the F2 to the least distorted in the F6, whilst the opposite was true for linkage group 7. Other changes were more minor – approximately 16% decrease in the total percentage of the distorted loci was observed for linkage groups 1 and 3, ∼12% increase for linkage groups 2 and 4, 7% drop for linkage group 5. The alleles associated with the pattern of deviation tend to be toward the maternal alleles in maps for F2 and F6. **Figure 6** shows the level of distortion at each locus (with *p*-values associated with the chi-square results from JoinMap) and the location of the distorted regions along the linkage groups.

### RIL Specific Features: Heterozygosity Retention

The average heterozygosity per locus in the F6 population was 5.4% (ranging between 0 and 18%).

**FIGURE 7:**
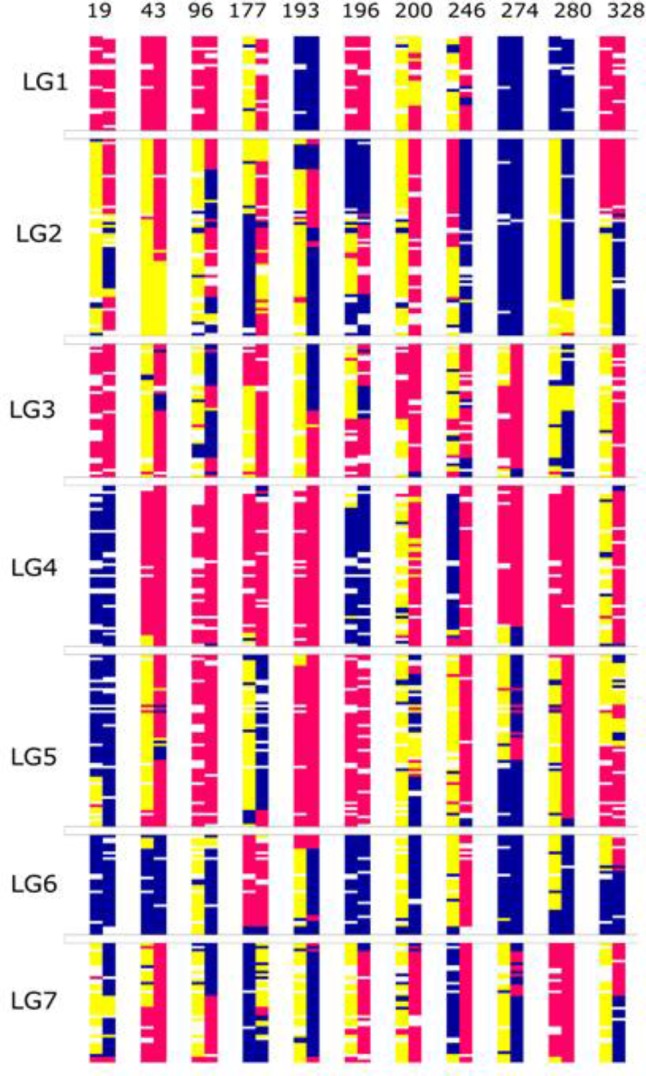
The graphical genotypes of the 356 common SNP loci in the eleven individuals that were common between the F2 and F6 map. The *x*-axis represents the individuals and the *y*-axis, the loci ordered by the F2 map position. Of the individual chromosomes, the first bar is from the F2 and the second bar is from the F6. The blue color represents allele from paternal parent (A), pink from maternal parent (B) and yellow (H) represents the heterozygous state.

The 41 F6 individuals in the population have been derived by single seed descent from the original F2 Biomass population comprising 325 individuals, originally described by Anhalt et al. (2008). One hundred and sixty-nine of these F2 individuals were used to create the dense SNP-based map, previously described by Velmurugan et al. (2016). Unfortunately, due to material availability at the time of the production of each of the maps, there were only eleven individuals from the current F6 population descending from the portion of the F2 Biomass population used to generate the map in the previous study. For these eleven lines exhibiting lineal descent, we examined the zygosity state of each linkage group of the F6 relative to its ancestral F2 line using the genotype data of the 356 common SNP loci present in both maps. In the majority of cases the state in the F6 was coherent with the state in the F2: the homozygous regions in the F2 were maintained in the F6, while heterozygous regions yielded homozygous regions derived from one of the two grandparental states, or (less frequently) remained heterozygous (**Figure 7**).

### RIL Specific Features: Crossover Frequency

Another key feature of RILs is the increased map resolution caused by the additional number of recombination events accumulated during continuous self-pollination. We used the graphical genotypes from the final map order results to manually count the number of crossover events and use this as an estimate to examine the number and distribution of the recombination events in the F6 population in comparison to the source F2 population.

In theory, assuming one crossover per chromosome arm per meiosis, the expected number of recombination events per genotype in the F6 generation is 27.5 (based on two chiasma per chromosome pair per meiosis). On average, from the graphical genotype results there were 17 observed recombination events per genotype and the map was based on a total of 705 recombination events (Supplementary Data File 3). This number is lower than expected, and to investigate its source we examined the graphical genotypes to quantify the presence of chromosomes derived from the original inbred parents of the population that had apparently been transmitted intact through the six generations of self-fertilization, and which were present in the homozygous state, precluding any further observable recombination. In the F6 genetic map, these homozygous non-recombinant parentally derived chromosome pairs were observed at a very high rate: out of 287 [41 (RILs) × 7 (chromosome pairs) = 287] chromosome pairs, 88 (31%) were non-recombinant (**Figure 4**). In the map of the F2 Biomass population 14% of chromosome pairs were in the above homozygous non-recombined state (167 (genotypes) × 7 (chromosome pairs) = 1,169 chromosome pairs, 164 of which were non-recombinant), and this represents the baseline below which the level could not fall. Completely heterozygous chromosome pairs occurred at a rate of 5% in the map of the F2 Biomass population (Supplementary Data Sheet 4). It is apparent that these chromosomes have frequently assorted without recombining over the subsequent rounds of self-fertilization. Similar to the F2, higher number of these non-recombinant chromosome pairs favored the maternal haplotype (52) than the paternal haplotype (36). On average 13 non-recombinant genotypes were observed per linkage group with the highest number seen in chromosome 1, 6 (16) and the least in chromosome 5 (8). There were no instances of completely heterozygous chromosomes in the F6 as opposed to the 5% seen in the F2.

## Discussion

Family-based linkage (FBL) studies continue to be extremely important in genetic analysis of perennial ryegrass. In many other systems, there has been a shift toward an increased use of genome wide association (GWAS) approaches for applications such as QTL mapping (Fe et al., 2015). This has been driven by a mixture of the continuously dropping costs associated with saturating genomes with markers, and the precision of GWAS, combined with the fact that the use of diverse germplasm panels allows the discovery of a greater number of QTLs relative to biparentally derived populations. However, the utility of GWAS in perennial ryegrass may be limited by rapid decay of LD in the species. For example Ponting et al. (2007) and Xing et al. (2007) have shown that LD decayed to background levels within 0.5 kb in eleven disease resistance genes among 20 diverse genotypes and between 0.5 and 3 kb in nutritive quality genes among diverse genotypes, respectively. In our own study findings presented here were very comparable to the findings of Ponting et al. (2007) and Xing et al. (2007). More recently, Fe et al. (2015), despite using nearly one million SNP markers on a GWAS panel of 1,000 F2 families, explained only a fraction of the observed variation for heading date in the population. They hypothesized that because LD extended only very short distances, this limited their ability to identify QTLs even with a high marker density.

Family-based linkage studies will continue to be essential in advancing the state of the art in gene discovery and trait dissection in perennial ryegrass as in many other species (Xu et al., 2017). Resources such as RIL populations can play a role in this by providing portable mapping populations that allow experiments to be carried out by multiple research groups, across multiple years, for traits that may have co-ordinated genetic control, although the restriction caused by limited diversity associated with FBL still applies in these cases.

Perennial ryegrass is an obligate outbreeder with a genetically determined self-incompatibility system, and thus the development of RILs is difficult in a normal background for the species. We have taken advantage of the existence of germplasm from the Teagasc forage breeding program in which the self-incompatibility system has broken down, allowing the development of inbred lines. Two of these lines were the grandparents of the F2 Biomass population described originally by Anhalt et al. (2008) and in which a high density SNP and PAV-based map was recently developed by Velmurugan et al. (2016). The current F6 population was developed via single seed descent from the larger F2 population. The ultimate goal of the work described here is the development of a reasonable sized (∼200) RIL population at the F8-F9 generation, that could be used as an immortalized high resolution mapping population for perennial ryegrass that could be distributed and maintained by seed. Development of RILs in perennial ryegrass is even more challenging than in inbreeding species, since inbreeding depression results in poor seed set. At the time of the study, the most advanced generation available from the long running initiative to create a RIL population in this species at Teagasc was the F6, and only 41 individuals of this generation were available. The main reason for carrying out the experiment was to provide the baseline information to decide whether further efforts should be expended on the development of the RILs, by testing whether the genomic constitution of the latest available generation was as per expectations. Despite that the population size was small, inter- and intra-chromosomal LD analysis suggested that the map order was robust. We have generated a robust genetic map for this RIL population with an ultra-high marker density. We can demonstrate that despite its small size the resolution power is very good and the size of the population will still allow QTL analysis. The required population size required for mapping comes down to the individual situation, the population, the marker systems, the resolution of the genetic map, ultimately its power. The principle of a RIL population is that it has a greater number of recombination events. Generally this means that populations of reasonable size from advanced RILs have much improved resolving power than similar sized populations from earlier generations. However, it also means that relatively small population sizes can still resolve markers. This presented RIL population is a small, portable, seed immortalized population which can be useful for indicative phenotyping. Despite the apparent suppression of recombination, the relatively small population size supports the generation of a linkage map with even slightly better accuracy and resolving power of a predecessor F2 population which is several times bigger.

Using the general approach described by Velmurugan et al. (2016) for the progenitor F2 population, we developed an ultra-high resolution genetic map of the population (6,072 SNPs and 22,080 PAVs, **Table 2**). This higher proportion of mapped PAV markers is similar to findings in maize where a five times greater amount of PAV markers compared to SNP markers was found (Elshire et al., 2011). Our here presented genetic map was developed by sequencing the 41 individuals on only a single channel on the Illumina HiSeq platform. Our expectations for the F6 population were that, relative to the F2 population, we should observe a decrease in heterozygosity and an increase in the observable number of recombination events in the progeny individuals based on the original grandparental haplotypes. These are, in fact, two of the key characteristics of RILs. Achievement of complete homozygosity renders the population “immortalized,” and theoretically, subsequent rounds of seed production for each individual genotype in the population can be performed via open pollination rather than single seed descent – radically enhancing the amount of seed that can be produced per line. Progress toward homozygosity in the population seems to be according to theoretical expectations, and we did not observe any unusual features, such as non-random retention of heterozygosity associated with specific genomic regions or related to specific RILs. This indicates that expectations of the rate of advance toward homozygosity in the RILs are being met. With a current level of heterozygosity of ∼5%, a further 2–3 rounds of self-pollination would render the population effectively homozygous, after which maintenance by open pollination would be feasible. One of the key goals of generating RILs is to make the individual genotypes in the population homozygous at all loci whilst representing a mosaic of original parental chromosomes by continuous self-pollination. The self-pollination causes the genotypes at each locus to gradually progress toward homozygosity.

**FIGURE 8:**
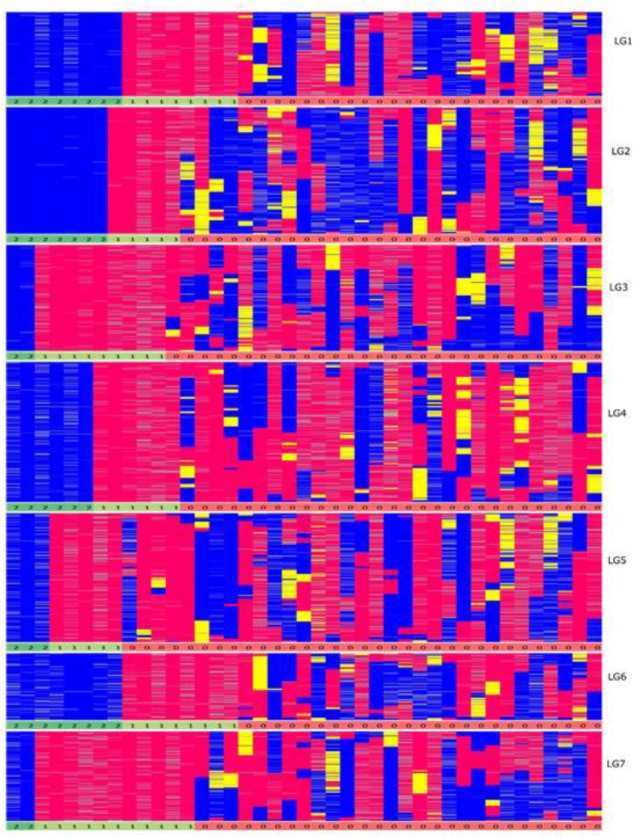
Graphical genotypes of the F6 framework SNPs ordered by completely homozygous to heterozygous genotypes for each linkage group (blue-paternal; pink-maternal; yellow-heterozygous) individually. The order of genotypes varies for each linkage group. The numbers below each linkage group represents if the individual chromosome is non-recombinant/recombinant. Number 1 (color coded as yellow green) – indicates non-recombinant for maternal allele, 2 (color coded as green) – indicates non-recombinant for paternal allele and 0 (color coded as red) – indicates recombinant.

One unusual feature that we noticed in the F6 population was the presence of a very high number of apparently non-recombined parentally derived chromosomes, present in the homozygous state. One third of chromosome pairs in the F6 population exhibited a homozygous state that reflected the fully intact version of the chromosome donated by one or the other biological parents of the original F1 genotype from which the population is derived, with significant skewing toward the maternal parent (**Figure 8**). These non-recombinant chromosome pairs have two possible sources – they are either derived from chromosome pairs that were already in this state at the F2 stage, and/or they are derived from completely heterozygous pairs at the F2 stage, and these have failed to recombine over several generations. However, a suppression of recombination could proof to be advantageous in some breeding schemes like in backcross breeding. In the first generations of backcross breeding suppression of recombination combined with marker-assisted selection would accelerate the complete restitution of the recurrent parent genome (Link and Melchinger, 1995). Since the developed RIL lines are a resource they can be incorporated into any forthcoming breeding scheme.

On chromosomes of the grandparents of the F2 population (the precursors of the RIL population) three maternal and four paternal pairs of nucleolus organiser regions (NORs) have been found (Anhalt et al., 2008). NORs tend to suppress recombination and hence regularly give rod rather than ring bivalents. Genetic maps of chromosomes with NORs should result in shorter linkage groups. Unfortunately we do not know which chromosomes with NORs in the F2 population and the resulting RIL population represent which linkage groups, but the shorter linkage groups could bear NORs. Whole linkage group arms in the RIL genotypes with almost no recombinants are candidate NOR arms. Possibly the top arm linkage group 3 is the most convincing for no recombinants and possibly linkage group 6. It can be speculated if the segregation distortion change from 96% in the F2 population to 8% in the F6 RIL population for linkage group 6 could be the polymorphic rDNA site becoming fixed (see rDNA *in situ* hybridization results of Anhalt et al., 2008).

The count for fixation of non-recombined parental homozygotes in the F2 generation was 14% of chromosome pairs. Hence in the F6 this value could not go below 14% as the F6 is derived from the F2 population. It seems that there is a tendency, through multiple rounds of self-pollination, of heterozygous chromosome pairs to assort without prior meiotic recombination, yielding non-recombined homozygous parental haplotypes in the next generation (rising to 31% in the F6). This apparent suppression of recombination between the parental chromosomes is also reflected in the lower than expected number of observed recombinants in the F6 generation. We do not know what mechanism might be behind this phenomenon. The phenomenon will ultimately impact on the overall map-resolution relative to what would be expected at this stage. It has been previously shown that in longer lived *Lolium* species lower chiasma frequencies have been observed compared to shorter lived species and that these lower chiasma frequencies in the longer lived *Lolium* species were associated with a higher phenotypic and genetic variance for traits under polygenic control (Rees and Dale, 1974). The original parental lines for the presented *Lolium* RILs were selected for maintainer character during inbreeding in the cytoplasmic male sterility (CMS) program. The question arises as to what effect, if any, this selection had on unusual behavior at meiosis. This implies suppressed recombination may have a role to play in the upkeep of essential traits like in our case of the maintainer character of the parental lines for CMS.

Whilst resolving power is an important feature of a map, the robustness of map order is also important. This feature of the map is based on a variety of factors, including the aforementioned total number of recombination events upon which the map is built, but also factors relating to the manner in which the map was created. Segregation distortion was a prevalent feature in the F2 map, with just over 30% of non-redundant framework markers exhibiting distortion. A similar overall level of distortion was observed in the F6 map, but there were striking changes in the distribution and extent of SD for chromosomes 6 and 7. These were, respectively, the most and least distorted chromosomes in the F2 genetic map, but the situation was reversed for the F6 genetic map, whereby chromosome 6 exhibited the least distortion and chromosome 7 the most. Such extensive shifts in SD are unlikely to be random, and may represent selection for and/or against certain allelic configurations in the four rounds of recurrent self-pollination.

The longer term goal is the production of a fully immortalized, seed distributable RIL population of perennial ryegrass. An ideal target would be a minimum of 200 genotypes at the F8 or F9 stage, since this population size would provide a good combination of resolving power and portability. The capability to maintain the population by open-pollination of individual genotypes of each RIL line in controlled isolation also opens up interesting possibilities such as experiments in which the unit of phenotyping is effectively a mini-sward or plot composed of genetically identical individuals. However, the relative robustness of the map in this study based on only 41 individuals means that there might be potential to exploit the current iteration of the genetic map. For instance, experiments in which phenotyping is prohibitively expensive and relates to complex quantitative inherited traits, or requires extensive replication might already benefit from the availability of a small population to investigate indicative segregation patterns for complex traits with the resolving power and apparent robustness available in this map.

The results from this study indicate that, apart from the recombinational suppression discussed above, the progress of the RIL population is largely as expected for the number of rounds of self-pollination. Given that a relatively small population size at the F6 generation is already capable of producing an apparently robust map, it seems reasonable to assume that the target of 200 RILs at the F8/F9 stage would generate a high quality, robust map, with a resolution capable of resolving the position of tens of thousands of SNP markers in a relatively economic fashion.

One problem associated with immortalization could be, however, seed yield since not all genotypes in the RILs behave the same in terms of seed yield. About 14% of the genotypes across all generations exhibited much higher seed yield than others. Seed yield is an important trait for any agriculturally important species, including perennial ryegrass. Experiments studying seed yield can be easily influenced by confounding factors including the environment and agricultural practices (Elgersma, 1990). Seed set in ryegrass is not 100% (Elgersma and Sniezko, 1988; Elgersma, 1990). This RIL population and its original F2 population could be very interesting to study seed yield in greater detail. In earlier work of Crowley and Rees (1968) it has been shown that selection for seed yield in accession of perennial ryegrass can be associated with unusual chromosome pairing. It would be worth investigating chromosome pairing behavior in the independent RIL lines.

Because of the intensive nature of RIL production in perennial ryegrass, our intention is to highlight this study to gain community support in the continued development of the germplasm described herein. We propose that the potentially large RIL population described above could be developed collaboratively across several interested research groups (each advancing a limited set of genotypes from the F5/F6 stage onward), producing a valuable resource that could act as an “integrator” across studies and groups, and which, allied to the integrative potential of the emerging genome sequences, would be a powerful resource for trait dissection and gene discovery in perennial ryegrass. To this end, we declare our intention to make the resources described in the study completely open access, and invite interested groups within the perennial ryegrass genetics and genomics community to contact us.

## Author Contributions

SB and JH-H conceived the population design. SB, DM, ML, and JV conceived the genetic mapping study. VC developed the inbred lines and SB the RIL lines. JV and DM prepared the genetic map. All authors contributed to the drafting of the manuscript, revised it critically, and approved the final version of the manuscript. All authors agreed on the final version of the manuscript. All authors agreed to be accountable for all aspects of the work regards to the accuracy of any part of the work.

## Conflict of Interest Statement

The authors declare that the research was conducted in the absence of any commercial or financial relationships that could be construed as a potential conflict of interest.

## Footnotes

1 https://www.ncbi.nlm.nih.gov/sra
