# Supplementary material for: An Immortalized Genetic Mapping Population for Perennial Ryegrass: A Resource for Phenotyping and Complex Trait Mapping
Source: Front Plant Sci. 2018 May 31;9:717. doi: 10.3389/fpls.2018.00717 (PMC5991167; doi:10.3389/fpls.2018.00717)
Supplement: Supplementary file 1 [file Table_1.docx]

**Supplementary Table 1:** Number of reads generated and number of reads mapped per individual of the F6 population and per barcode used.

| F6 individual ID | Barcodes used | Total no. of reads | Total no. of reads mapped |
| --- | --- | --- | --- |
| F6_007 | AGTTAAT | 3734004 | 1892608 |
| F6_007 | GGAACGA | 2873364 | 1469888 |
| F6_019 | CCACCA | 3228690 | 1790440 |
| F6_043 | GCTAACA | 3883570 | 1843324 |
| F6_067 | TCCGCA | 3508444 | 1752120 |
| F6_067 | AAGACGCT | 3463530 | 1780730 |
| F6_078 | TGACGCCA | 2877080 | 1497652 |
| F6_078 | GGCTTA | 2946266 | 1579986 |
| F6_078 | AGTCAAGA | 3588204 | 1851404 |
| F6_082 | GCGCTCA | 2780564 | 1671700 |
| F6_082 | ACTGCT | 3318326 | 1999102 |
| F6_082 | CAGGCCACT | 2676468 | 1599468 |
| F6_089 | AATCG | 3426648 | 1658268 |
| F6_089 | CGCTCA | 2470628 | 1292496 |
| F6_094 | ATCATACCT | 3868612 | 2015884 |
| F6_096 | CTTCCTCT | 3594044 | 1826582 |
| F6_104 | GGAG | 2949452 | 1644198 |
| F6_104 | TTGCGTCT | 3211508 | 1770454 |
| F6_111 | TCTCA | 3861838 | 2099668 |
| F6_111 | CCGAACA | 2929892 | 1627822 |
| F6_118 | AACGCACATT | 2972880 | 1659176 |
| F6_166 | ATGG | 3218304 | 1654978 |
| F6_177 | CCATCCACT | 3206816 | 1564532 |
| F6_193 | GACAG | 3054350 | 1623398 |
| F6_193 | CATCTGCCG | 2798222 | 1529008 |
| F6_196 | GAAGTG | 2986164 | 1640768 |
| F6_200 | TTCGTT | 3190950 | 1618114 |
| F6_215 | CCACTCA | 2683846 | 1521374 |
| F6_215 | AGTGTTAA | 3554896 | 1934752 |
| F6_217 | TAGATGA | 2979752 | 1743442 |
| F6_229 | ACCA | 3210690 | 1929296 |
| F6_229 | CTCACT | 3436894 | 2047934 |
| F6_230 | CCTG | 3221504 | 1541600 |
| F6_246 | GACATCCA | 3552648 | 1619604 |
| F6_253 | GGTATA | 2873952 | 1838354 |
| F6_256 | GCAAGCCAT | 3128914 | 1732108 |
| F6_256 | TCAT | 3979442 | 2163914 |
| F6_257 | TCACGGAAG | 3127990 | 1805074 |
| F6_274 | CGCAACCAGT | 2409004 | 1498802 |
| F6_274 | TTGTAG | 3842066 | 2171800 |
| F6_276 | AGAATGA | 3490392 | 2087450 |
| F6_276 | GGAAGACAT | 2705574 | 1617430 |
| F6_280 | ATTACA | 3930948 | 2051226 |
| F6_283 | CGACG | 2947354 | 1475654 |
| F6_283 | GCGCCACT | 2680618 | 1342304 |
| F6_294 | GACTCGG | 3141330 | 1757962 |
| F6_295 | GTGCACCA | 3015728 | 1503300 |
| F6_295 | GATGA | 3423352 | 1699090 |
| F6_307 | TATTCGCAT | 3288310 | 1544762 |
| F6_324 | TCTTGG | 3301002 | 1553738 |
| F6_324 | CTAAGCA | 3352510 | 1585200 |
| F6_328 | TATCA | 3286464 | 1761192 |
| F6_329 | AGGAG | 3068432 | 1585560 |
| F6_342 | GAGCGAA | 3165078 | 1608434 |
| F6_355 | TTGACCAG | 3109682 | 1673568 |
| F6_366 | ATAGAT | 3439226 | 1601744 |
| F6_368 | GGTGT | 2567078 | 1408388 |
| F6_368 | ATGAGCAA | 3370410 | 1827330 |
| F6_370 | AATTAG | 3636746 | 1847228 |
| F6_371 | CTCGCGG | 2454668 | 1241052 |
| F6_371 | TAGCAG | 3253726 | 1684754 |
