# Supplementary material for: An Immortalized Genetic Mapping Population for Perennial Ryegrass: A Resource for Phenotyping and Complex Trait Mapping
Source: Front Plant Sci. 2018 May 31;9:717. doi: 10.3389/fpls.2018.00717 (PMC5991167; doi:10.3389/fpls.2018.00717)
Supplement: Supplementary file 2 [file Data_Sheet_2.docx]

Supplementary data file 2: Filtering criteria used to identify the final set of high confidence SNPs

| Total Reads/Read Pairs | 226,467,750 / 113,233,875 |
| --- | --- |
| ~Reads/Read pairs per individual (million) | 3.1/1.55 |
| Reads mapped to reference | 119,392,038 |
| Reads failed to map to reference | 98370816 |
| Reads supressed due to mapping to multiple positions | 8,704,896 |
| Reported SNPs from Varscan (default settings) | 98042 |
| SNPs after filtering to exclude monomorphic and >35% missing values | 16,417 |
| Biallelic/Triallelic SNPs | 16,323/94 |
| Biallelic SNPs grouping into LGs at 0.2/18 (RF/LOD) | 9,817 |
| Grouped at LOD >=6 using ‘independence LOD’ function of JoinMap 4.1 | 7806 |
| High confidence SNPs with read depth >20 | 6,072 |

In total, the paired end Illumina sequencing yielded 226,467,750 reads (113,233,875 read pairs). After de-multiplexing, on average 3.1 million reads (~1.5 million read pairs) per individual were obtained. In total 119,392,038 (53%) of the total 226,467,750 reads  aligned to the reference set allowing three mismatches, 8,704,896 reads (4%) were suppressed as they mapped to more than one region in the reference set and 98,370,816 reads (43%) failed to align. Using VarScan.v2.2.11 with the default settings, 98,042 SNP variants were reported. The filtering criteria adopted to exclude monomorphic markers and markers with large number of missing values (>35%) yielded 16,417 SNPs. Out of these 16,323 SNPs were bi-allelic and 94 of them were tri-allelic. Of the 16,323 biallelic SNP variants, the majority were of transition types: C/T (30%) and A/G (30%) and the remaining were of transversion types: C/G (15%), G/T (10%), A/C (9%) and A/T (6%) respectively. 9,817 out of 16,323 SNPs grouped into seven linkage groups at a recombination fraction (RF) vs LOD score threshold of 0.2/18. For the framework map only 7806 markers that grouped at LOD >=6 using ‘independence LOD’ function of JoinMap 4.1 were carried forward. An additional round of filtering for SNPs from alignments with a read depth of > 20 reduced the number to a set of 6072 high confidence SNPs.
