# Supplementary material for: An Immortalized Genetic Mapping Population for Perennial Ryegrass: A Resource for Phenotyping and Complex Trait Mapping
Source: Front Plant Sci. 2018 May 31;9:717. doi: 10.3389/fpls.2018.00717 (PMC5991167; doi:10.3389/fpls.2018.00717)
Supplement: Supplementary file 3 [file Data_Sheet_3.docx]

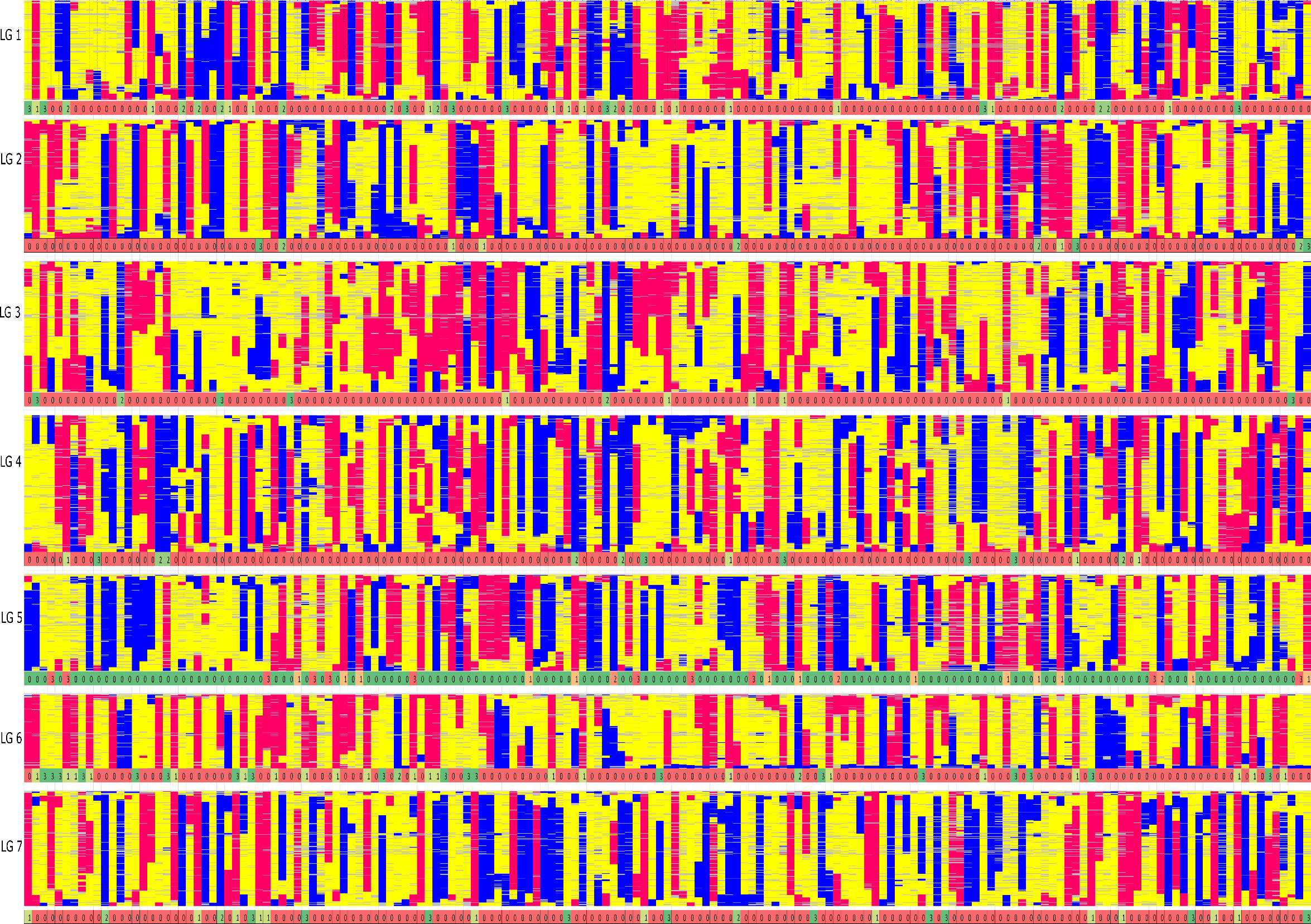


**Supplemental Data File 3L F2 Graphical Genotypes and Crossover:** Graphical genotypes of the F2 framework SNP map. The x-axis represents the error corrected genotype calls of the 167 F2 individuals and the y-axis consist of markers ordered by chromosomal map position based on the final maximum likelihood map order of the framework SNP markers. The colours ‘pink’ represent homozygous alleles from the maternal parent, ‘blue’ represent homozygous alleles from paternal parent, ‘yellow’ as heterozygous alleles and grey as missing data. The numbers below each linkage group represents if the individual chromosome is non-recombinant / recombinant. Number 1 (colour coded as yellow-green) – indicates non-recombinant for maternal allele, 2 (colour coded as green) – indicates non- recombinant for paternal allele and 3 (colour coded as dark green)– indicates non-recombinant for heterozygous allele and 0 (colour coded as red) indicates recombinant chromosomes.
